# Dietary Flavonoid Intake and Smoking-Related Cancer Risk: A Meta-Analysis

**DOI:** 10.1371/journal.pone.0075604

**Published:** 2013-09-19

**Authors:** Hae Dong Woo, Jeongseon Kim

**Affiliations:** Molecular Epidemiology Branch, National Cancer Centre, Goyang-si, Korea; University of Louisville, United States of America

## Abstract

**Purpose:**

To systematically investigate the effects of dietary flavonoids and flavonoid subclasses on the risk of smoking-related cancer in observational studies.

**Methods:**

Summary estimates and corresponding standard errors were calculated using the multivariate-adjusted odds ratio (OR) or relative risk (RR) and 95% CI of selected studies and weighted by the inverse variance.

**Results:**

A total of 35 studies, including 19 case-controls (9,525 cases and 15,835 controls) and 15 cohort studies (988,082 subjects and 8,161 cases), were retrieved for the meta-analysis. Total dietary flavonoids and most of the flavonoid subclasses were inversely associated with smoking-related cancer risk (OR: 0.82, 95% CI: 0.72-0.93). In subgroup analyses by cancer site, significant associations were observed in aerodigestive tract and lung cancers. Total dietary flavonoid intake was significantly associated with aerodigestive tract cancer risk (OR: 0.67, 95% CI: 0.54-0.83) marginally associated with lung cancer risk (OR: 0.84, 95% CI: 0.71-1.00). Subgroup analyses by smoking status showed significantly different results. The intake of total flavonoids, flavonols, flavones, and flavanones, as well as the flavonols quercetin and kaempferol was significantly associated with decreased risk of smoking-related cancer in smokers, whereas no association was observed in non-smokers, except for flavanones. In meta-analysis for the effect of subclasses of dietary flavonoids by cancer type, aerodigestive tract cancer was inversely associated with most flavonoid subclasses.

**Conclusion:**

The protective effects of flavonoids on smoking-related cancer risk varied across studies, but the overall results indicated that intake of dietary flavonoids, especially flavonols, was inversely associated with smoking-related cancer risk. The protective effects of flavonoids on smoking-related cancer risk were more prominent in smokers.

## Introduction

Flavonoids are polyphenolic compounds that are abundant in fruits and vegetables. High intake of fruits and vegetables is associated with beneficial health effects, and these effects have been attributed in part to their high content of flavonoids. The World Cancer Research Fund (WCRF) and the American Institute of Cancer Research (AICR) reported summary estimates of the effects of fruit and vegetable consumption on cancer risk [1]. The consumption of fruits and vegetables most likely protects against cancers of the mouth, pharynx, larynx, esophagus, and stomach; the risk of lung cancer was only associated with fruit consumption. These cancers are smoking-related cancers according to the International Agency for Research on Cancer (IARC) Monograph on tobacco smoking. Smoking is classified as the cause of cancers of the lung, oral cavity, nasal and paranasal sinuses, pharynx, larynx, esophagus, kidney, liver, uterine cervix, stomach, bladder, pancreas, as well as myeloid leukemia [2]. Adverse effects of flavonoids in human health are rare, but the potential for detrimental health effects is based on in vitro studies in which pro-oxidant activities have been observed [3,4]. However, flavonoids are known to have powerful antioxidant, anti-inflammatory, and anti-tumor activities against carcinogens [5,6,7]. Tobacco contains various carcinogens that can induce free radicals and cause gene mutations and the formation of DNA [8]. Thus, dietary flavonoids may play a role in protecting against smoking-related cancers.

The effects of dietary flavonoids on stomach and colorectal cancer were investigated using published studies [9] because flavonoids have been consistently found to be inversely associated with stomach and colorectal cancers in in-vitro studies [10,11,12]. However, based on a previous meta-analysis, no clear evidence supports that dietary flavonoids are associated with a reduced risk of stomach and colorectal cancers. The health effects of flavonoids and their subclasses might differ by cancer site. The cancer site that is affected by carcinogens such as tobacco smoking might be closely related to intakes of dietary flavonoids. Thus, a meta-analysis of published case-control and cohort studies was performed to calculate summary estimates of the effects of dietary flavonoid and their subclasses on smoking-related cancer risk.

## Methods

### Study selection

A systematic search for relevant studies published through October 31, 2012 was conducted with PubMed using the terms (cancer risk) and (flavonoid or flavonol or quercetin or kaempferol or myricetin or isorhamnetin or flavone or luteolin or apigenin or flavanone or eriodictyol or hesperetin or naringenin or flavan-3-ol or catechin or epicatechin or theaflavin or anthocyanidin or cyanidin or delphinidin or malvidin or pelargonidin or peonidin or petunidin or isoflavones). The inclusion criteria were as follows: (1) the original article described a case-control or cohort design; (2) the article reported the intake of either dietary flavonoids or subclasses of flavonoids; (3) the article reported the risk of smoking-related cancers that were defined according to the IARC monograph (cancers included those of the oral cavity, paranasal sinuses, nasal cavity, larynx, pharynx, lung, esophagus, stomach, liver, pancreas, kidney, bladder, and uterine cervix, as well as myeloid leukemia); and (4) the article reported 95% confidence intervals (CI) with adjusted odds ratios (OR) or relative risks (RR) for smoking-related cancer risk in subjects with the highest dietary flavonoid intake compared with those with the lowest dietary flavonoid intake.

### Data collection

Data on the authors, publication year, cancer site, country in which the study was performed, study design, study period, dietary assessment method, reported flavonoid types, included subclasses for calculation of total flavonoid intake, controlled confounders, and multivariate-adjusted OR/RR and 95% CI were collected for the meta-analysis. If the study reported only the subgroup analyses by sex, viral infection status, or ethnicity, then the summary estimate of the subgroup was used for the meta-analysis to include an example from each article. However, the articles that reported individual risk estimates by cancer sites but not an all-cancer risk were used only in the subgroup meta-analysis that was conducted by cancer site. If multiple articles reported the risk of the same cancer site from the same cohort data, then the most recently published data were selected.

### Statistical analysis

All statistical analyses were performed using the Stata software package (version 10, College Station, TX). Summary estimates and the corresponding standard errors were calculated using the multivariate-adjusted OR/RR and 95% CI of selected studies and weighted by the inverse variance. The heterogeneity was tested using the I^2^ test and Q-test based on the χ^2^ statistic, considering significant statistical heterogeneity as p < 0.1. Subgroup analyses were conducted by study design, sex, and smoking status. Publication bias was examined using Begg’s tests. A subgroup analysis by smoking status was performed when the studies reported the risk in both smokers and non-smokers because the effect of smoking on cancer risk can vary by cancer site. Sensitivity analyses were performed to test the robustness of the results of the combined effects. Based on the heterogeneity of the included studies, fixed or random effects models were selected to calculate the pooled effect measures.

## Results

**Figure 1:**
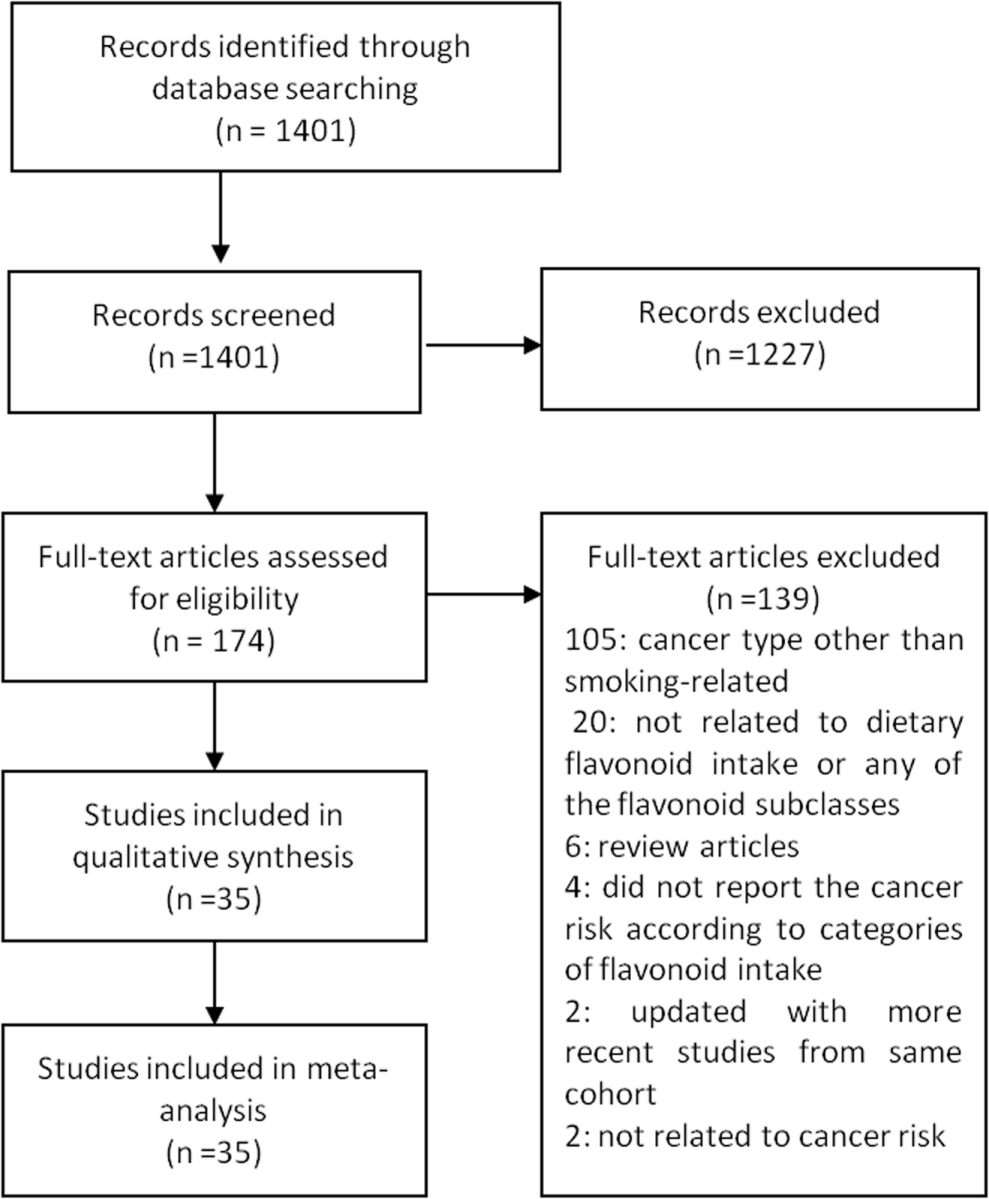
Flow diagram of study selection.

A total of 1401 studies were examined, and the 174 studies that remained after excluding articles based on the titles and abstracts were further reviewed; 139 studies were excluded for the following reasons: 106 studies included a cancer type that was not smoking-related; 20 studies were not relevant for dietary flavonoid intake or any of the flavonoid subclasses; 6 studies were review articles; 4 studies did not report the cancer risk according to categories of flavonoid intake; 2 studies were updated with more recent studies from the same cohort; and 2 studies were not related to cancer risk (Figure 1). Finally, 19 case-control studies [13,14,15,16,17,18,19,20,21,22,23,24,25,26,27,28,29,30,31] and 15 cohort studies [32,33,34,35,36,37,38,39,40,41,42,43,44,45,46] were selected for the meta-analysis (Tables 1, 2). Begg’s tests for publication bias showed non-significant results.

**Table 1:** Case-control studies on dietary flavonoids and risk of smoking related cancer.

First author [Ref no.] Year	Cancer site	Country	Study period	Case N / Control N	Dietary assessment method	Reported flavonoids	Included subclasses for total flavonoids	Intake comparison, High vs. low (mg/d)	Controlled confounders
Garcia-Closas [13] 1998	Lung	Spain	1989-1992	103/206	Diet history	Q, K, L		Median, Q: 6.58 vs. 2.5, K: 1.24 vs. 0.34, L: 0.02 vs. 0.00	Age, area of residence, and hospital and adjusted for smoking status (never-smokers, former smokers, current smokers), total pack-years smoked, vitamin E and vitamin C intake, total carotenoid intake (a-carotene, P-carotene, lutein, and lycopene), and intake of myricetin and other specific flavonoids as indicated.
Garcia [14] 1999	Bladder	Spain	1983–1986	495/1112	Diet history	Q, K, M, L		Q4 vs. Q1, Mean (SD), Case: Q: 4.8 (3.2), K: 0.97 (1.15), M: 0.23 (0.35), L: 0.39 (1.34) Control: Q: 4.8 (3.3), K: 1.03 (1.18), M: 0.21 (0.34), L: 0.38 (1.29)	Matched by gender, age, area of residence, and hospital. Adjusted for smoking status (never-smokers, former smokers, current smokers), total pack-years smoked, occupational exposure (no/yes), total energy intake (by residual method), vitamin E intake, saturated fatty acid intake, and intake of other specific carotenoids or other specific flavonoids.
Garcia-Closas [15] 1999	Gastric	Spain	1987-1989	354/354	Diet history	Q, K, M		Q4 vs. Q1, Mean (SD), Case: Q: 7.1 (6.5), K: 1.2 (1.9), M: 0.65 (1.17), Control: Q: 8.6 (9.0), K: 1.4 (2.0), M: 0.70 (1.42)	Total energy intake, intake of nitrites, nitrosamines, vitamin C, total carotenoids (α-carotene, β-carotene, lutein, and lycopene) and other specific favonoids (quercetin, kaempferol, myricetin, and luteolin).
Stefani [16] 1999	Lung	Uruguay	1993-1997	541/540	FFQ	T, Q, K	F1, F2	Q4 vs. Q1 Case: Mean (SD) Q: 5.2(5.3), K 2.1 (3.9) Control: Q: 6.8 (7.3), K 3.2 (5.6)	Age, residence, urban/rural status, education, family history of a lung cancer in 1st-degree relative, BMI, smoking (pack-yr), and total energy and total fat intake
Marchand [17] 2000	Lung	US (Hawaii)	1992-1997	582/582	FFQ	T, Q, K, M, H, N	Q, K, M, H, N	Q4 vs. Q1 (T3 vs. T1 for N)	Matched by age, sex, and ethnicity. Adjusted for smoking status, duration, (duration)^2^, number of cigarettes smoked per day, and b-carotene and saturated fat intakes
Lagiou [18] 2004	Lung	Greece	1987-1989	154/145	FFQ	F1, F3, F4		F1: per 5.0, F3: per 37.0, F4: per 5.7	Age, total energy intake, smoking status (ever vs. never smokers), and pack-years (among smokers), fruit and vegetable consumption.
Lagiou [19] 2004	Stomach	Greece	1981-1984	110/100	FFQ	F1, F2, F3, F4, An, I		F1: per 10.0, F2: per 0.3, F3: per 19.8, F4: per 135.1, An: per 40.4, I: per 2.0.	Age, gender, total energy intake, place of birth, BMI, height, years of education, smoking habits and duration of smoking, alcohol consumption, and fruit and vegetable consumption.
Schabath [20] 2005	Lung	US	1995-2003	1674/1735	FFQ	I		≥0.997 vs. ≤0.307	Age, sex, ethnicity, smoking status, cigarettes smoked per day, years of smoking, education, income, BMI, and total energy, where appropriate
Bosetti [21] 2007	Renal	Italy	1992-2004	767/1534	FFQ	T, F1, F2, F3, An, I	F1, F2, F3, F4, An, I	T: >180.9 vs. ≤80.6, F1: >29.9 vs. ≤13.3, F2: >0.6 vs. ≤0.3, F3: >57.8 vs. ≤9.6, F4: >90.6 vs. ≤21.3, An: >32.4 vs. ≤5.5, I: >32.6 vs. ≤14.8	Matched by age, sex, and study center. Adjusted for period of interview, education, tobacco smoking, alcohol drinking, BMI, occupational physical activity, family history of kidney cancer, and total energy intake
Garavello [22] 2007	Laryngeal	Italy	1992 - 2000	460/1088	FFQ	T, F1, F2, F3, F4, An, I	F1, F2, F3, F4, An, I	T: >221.8 vs. ≤95.5, F1: >33.7 vs. ≤16.8, F2: >0.7 vs. ≤0.3, F3: >49.2 vs. ≤7.7, F4: >110.4 vs. ≤31.2, An: >41.1 vs. ≤8.6, I: >32.6 vs. ≤14.7	Matched by age, sex, year of interview and area of residence. Adjusted for study centre, education, alcohol consumption, tobacco smoking, BMI, occupational physical activity and non-alcohol energy intake
Rossi [23] 2007	Esophageal	Italy	1992 - 1997	304/743	FFQ	T, F1, F2, F3, F4, An	F1, F2, F3, F4, An	T: >217.4 vs. ≤96.5, F1: >31.9 vs. ≤15.9, F2: >0.7 vs. ≤0.3, F3: >58.8 vs. ≤6.4, F4: >109.1 vs. ≤32.6, An: >41.2 vs. ≤8.1	Matched by age, sex, year of interview and area of residence. Adjusted for study centre, education, alcohol consumption, tobacco smoking, BMI and energy intake
Rossi [24] 2007	Oral cavity and pharyngeal	Italy	1992 - 2005	805/2081	FFQ	T, F1, F2, F3, F4, An, I	F1, F2, F3, F4, An, I	T: >204.0 vs. ≤83.5, F1: >29.9 vs. ≤13.9, F2: >0.67 vs. ≤0.3, F3: >67.0 vs. ≤10.2, F4: >99.6 vs. ≤23.3, An: >33.2 vs. ≤5.3, I: >32.5 vs. ≤14.7, (Sex and smoking status: per 1SD increment)	Matched by age, sex, and study center. Adjusted for tobacco smoking, alcohol drinking, education, BMI, and non–alcohol energy intake
Cui[25] 2008	Lung	US	1999-2004	558/837	FFQ	T, Q, K, M, H, N2, C, E	F1, F2, F3, F4, An	T: ≥90 vs. <30, Q: ≥7.5 vs. <2.5, K: ≥1.5 vs. <0.5, M: ≥0.6 vs. <0.2, H: ≥30 vs. <10, N2: ≥15 vs. <5, C: ≥3 vs. <1, E: ≥9 vs. <3	Matched by age, sex. Adjusted for race-ethnicity, years of schooling, smoking status, pack-years of tobacco smoking, and daily energy intake.
Lagiou [26] 2008	Liver	Greece	1995-1998	339/360	FFQ	T, F1, F2, F3, F4, An, I	F1, F2, F3, F4, An, I	T: >358.1 vs. ≤145.8, F1: >37.3 vs. ≤21.6, F2: >1.16 vs. ≤0.25, F3: >120.6 vs. ≤57.0, F4: >66.3 vs. ≤25.3, An: >152.7 vs. ≤10.2, I: >0.32 vs. ≤0.03	Age, sex, education, tobacco smoking, and total energy intake
Bobe [27] 2009	Esophageal	US	1986-1989	161/678	FFQ	T, F1, F2, F3, F4, An, I	F1, F2, F3, F4, An, I	(mg/1000kcal) T: >107 vs. <43, F1: >15.9 vs. <6.89, F2: >4.41 vs. <2.08, F3: >26.2 vs. <9.30, F4: >60.6 vs. <10.3, An: >4.73 vs. <1.45, I: >0.019 vs. <0.005	Smoking duration and intensity, geographical area, age, BMI, hot tea consumption, hard liquor consumption, beer consumption, moonshine consumption (for black men), red wine consumption, white wine consumption (except for ESCC in white men), caloric intake, education (for black men) and income
Rossi [28] 2010	Stomach	Italy	1997-2007	230/547	FFQ	F1, F2, F3, F4, An, I		F1: >32.3 vs. <13.2, F2: >0.7 vs. <0.3, F3: >56.8 vs. <12.9, F4: >79.2 vs. <21.6, An: >21.5 vs. <6.2, I: >34.3 vs. <15.0, P: >373.0 vs. <339.6.	Age, sex, education, year of interview, BMI, tobacco smoking, and total energy intake.
Ekstrom [29] 2011	Stomach (cardia & non cardia)	Sweden	1989-1995	C81, Non420/1116	FFQ	Q		>11.89 vs. <3.88.	Age, sex, socioeconomic status, number of siblings, BMI, smoking and energy and salt intake.
Christensen [30] 2012	Lung	Canada	1996-1997	f 399/ 574, m 662/851	FFQ	T, F1, F2, F3, F4, An	F1, F2, F3, F4, An	F1: f <7.6 vs. ≥16.6, m: <11.7 vs. ≥24.3, F2: f <0.6 vs. ≥1.4, m <0.7 vs. ≥2.1, F3: f <18.7 vs. ≥50.3, m: <20.8 vs. ≥64.6, F4: f <8.7 vs. ≥249.1, m <12.6 vs. ≥271.0, An: f <8.0 vs. ≥15.4, m <6.6 vs. ≥23.1	Age, sex, number of school years, mean census tract family income, ethnic group, respondent status, comprehensive smoking indicator, occupational exposure to carcinogens, BMI, number of alcoholic drinks/day and total energy intake
Rossi [31] 2012	Pancreas	Italy	1991-2008	326/652	FFQ	F1, F2, F3, F4, An		F1: >31.1 vs. ≤13.9, F2: >0.7 vs. ≤0.3, F3: >61.0 vs. ≤9.6, F4: >97.9 vs. ≤23.8, An: >31.1 vs. ≤4.5	Matched by age, sex, and center of study. Adjusted for year of interview, education, history of diabetes, tobacco smoking, alcohol drinking and non-alcohol energy intake

T: total flavonoid intake, Q: quercetin, K: kaempferol, M: myricetin, L: luteolin, H: hesperetin, N: naringenin, C: catechin, E: epicatechin, F1: flavonols, F2: flavones, F3: flavanones, F4: flavan-3-ols, An: anthocyanidins, I: isoflavones, P: proanthocyanidins, Q4: highest quartile, T3: highest tertile

**Table 2:** Cohort studies on dietary flavonoids and risk of smoking related cancer.

First author [Ref no.] year	Cancer site	Country	Follow-up (y)	Case (n)	Dietary assessment method	Reported flavonoids	Included subclasses for total flavonoids	Intake comparison, High vs. low (mg/d)	Controlled confounders
Arts[32] 2001	Lung	Mixed	<10	42	Diet history	F4		123.7 vs.25.2 (mean)	Age, physical activity, total energy intake, alcohol intake, smoking status, pack-years of smoking, BMI, coffee, fiber, vitamin C, vitamin E, β-carotene
Hirvonen [33] 2001	Stomach, Urothelial^a^	Finland	6.1 (median)	S 111/U 156	Diet history	T	F1, F2	16.3 vs. 4.2 (median)	Age and supplementation group.
Arts [34] 2002	Pancreas, Lung, Kidney and renal pelvis, Bladder	US	<13	P 130/L 549/K 114/ B 103	FFQ	F4		75.1 vs. 3.6 (mean)	Age, total energy intake, BMI, waist-to-hip ratio, physical activity, pack-years of smoking, smoking status, number of years since quit smoking, alcohol intake, and total fruit and vegetable consumption
Knekt [35] 2002	Stomach, Lung, Urinary organs	Finland	<30	S 74/ L 169/ U 81	Diet history	T, Q, K, M, H, N	F1, F2, F3	T: f >39.5 vs. 8.5, m 26.9 vs. 4.3, Q: f >4.7 vs. <1.8, m 3.9 vs. < 1.5, K: f >0.9 vs. <0.2, m 0.8 vs. 0.1, M: f >0.2 vs. <0.03, m 0.11 vs. 0.06, H: f >26.8 vs. <3.2, m 15.4 vs. 0, N: f >7.7 vs. <0.9, m 4.7 vs. 0	Age, sex, geographic area, occupation, smoking, and BMI.
Wright [36] 2004	Lung	Finland	11.3 (median)	1787	FFQ	T	F1, F4	Q4 vs. Q1 (IQR: 12.76)	Energy intake, age, number of cigarettes smoked per day, number of years of smoking, intervention assignment, BMI, and educational level.
Nothlings [37] 2007	Pancreas	US (multi-ethnic)		529	FFQ	F1, Q, K, M		Q5 vs. Q1	Age at cohort entry, history of diabetes mellitus, family history of pancreatic cancer, BMI, smoking status, pack-years of smoking, processed and red meat intake, and energy intake
Bobe [38] 2008	Pancreas	Finland	16.1 (median)	306	FFQ	T, F1, F2, F4, Q, K, M, L, A, C, E	F1, F2, F4		Age at randomization, years of smoking, total number of cigarettes per day, self-reported history of diabetes mellitus, and energy-adjusted saturated fat intake
Cutler [39] 2008	Lung	US	<18	849	FFQ	T, F1, F2, F3, F4, An, I	F1, F2, F3, F4, An, I, P	T: 680.0 vs. 91.0, F1: 23.2 vs. 3.9, F2: 1.75 vs. 0.11, F3: 107.2 vs. 7.4, F4: 314.6 vs. 4.1, I: 1.83 vs. 0.07 (mean)	Age, energy, education level, race, BMI, multivitamin use, activity level, smoking history, and pack years
Mursu [40] 2008	Lung	Finland	16.2 (mean)	62	Food recording	T, F1, F2, F3, F4, An	F1, F2, F3, F4, An,	T: 415.8 vs. 0.0 (energy adjusted mean)	Age and examination years, BMI, smoking status, pack-years of smoking, physical activity, intakes of alcohol, total fat and saturated fat, and energy adjusted intake of fiber, vitamin C and E.
Kurahashi [41] 2009	Liver	Japan	11.8 (mean)	m69/f32	FFQ	I (gen)		≥20.0 vs. <12.0 (m), ≥19.6 vs. <12.2 (f)	Age, area, HCV, HBsAg, smoking status, alcohol consumption, and intake of coffee and vegetables (+ menopausal status for women).
Wang [42] 2009	Lung	US	11.5 (mean)	241	FFQ	T, Q, K, M, L, A	F1, F2,	T: 47.44 vs. 8.88, Q: 32.79 vs. 6.49, K: 13.06 vs. 0.86, M: 2.83 vs. 0.15, L: 0.20 vs. 0.01, A: 1.35 vs. 0.13 (median)	Age, race, total energy intake, randomized treatment assignment, smoking, alcohol use, physical activity, postmenopausal status, hormone replacement therapy use, multivitamin use, BMI, family history of colorectal cancer, ovary cancer, and breast cancer, and intake of fruit and vegetables, fiber, folate, and saturated fat.
Bertoia [43] 2010	Renal	Finland	6.1 (median)	255	FFQ	T, Q, K, M, C, E	F1, F4	T: 39.66 vs. 4.76, Q: 13.20 vs. 3.34, K: 4.26 vs. 0.13, M: 1.94 vs. 0.24, C: 7.37 vs. 0.18, E: 12.85 vs. 0.21 (mean)	Age, BMI, education level, measured systolic and diastolic blood pressure, self-reported history of hypertension, leisure-time physical activity, years of smoking, total number of cigarettes per day, trial intervention group, and alcohol consumption, total energy intake
Shimazu [44] 2010	Lung	Japan	8 (median)	m481/f178	FFQ	I		Q4 vs. Q1	Age, study area, smoking status, alcohol consumption, menopausal status in women, and total intake of vegetables, fruit, and fish.
Hara [45] 2012	Stomach	Japan		1249	FFQ	I (genistein)		Q4 vs. Q1, (median), 42.3 vs. 9.2 (m), 41.8 vs. 9.4 (f)	Age, public center area, BMI, smoking status, ethanol intake, family history of gastric cancer, vegetable, fruit, fish, salt, and total energy intake.
Zamora-Ros [46] 2012	Stomach	10 Europe	11.0 (mean)	683	FFQ, food records	T, F1, F2, F3, F4, An, I	F1, F2, F3, F4, An, I, P, Th	T: >595.5 vs. <200.4, F1: >33.7 vs. <15.0, F2: >4.6 vs. <1.2, F3: >29.2 vs. <6.1, F4: >199.9 vs. <26.1, An: >32.8 vs. <11.7, I: >1.1 vs. <0.3	Age, educational level, smoking status, physical activity, BMI, alcohol and energy intake, and daily consumption of fruit, vegetables, red and processed meat

a Lung and renal cell cancer were omitted in the analysis because updated articles were published using same cohort data. T: total flavonoid intake, Q: quercetin, K: kaempferol, M: myricetin, L: luteolin, A: apigenin, H: hesperetin, N: naringenin, C: catechin, E: epicatechin, F1: flavonols, F2: flavones, F3: flavanones, F4: flavan-3-ols, An: anthocyanidins, I: isoflavones, P: proanthocyanidins, Th: theaflavins, Q4: highest quartile, Q5: highest quintile.

Table 3 shows the meta-analysis of the risk of smoking-related cancer in people with the highest intake of total dietary flavonoids compared with those with the lowest intake of total dietary flavonoids. Total dietary flavonoids were inversely associated with smoking-related cancer risk. However, in the subgroup analysis conducted by study design, this association was observed only in the case-control studies. Intake of total dietary flavonoids was inversely associated with smoking-related cancer risk among smokers, but the associations were not significant for the non-smokers. The summary estimate calculated for the association between total dietary flavonoids and aerodigestive tract cancer risk was significant (OR: 0.67, 95% CI: 0.54-0.83). The association with lung cancer risk was only marginally significant (OR: 0.84, 95% CI: 0.71-1.00).

**Table 3:** Summary estimates for the effect of total dietary flavonoids on risk of smoking-related cancer ^**a**^.

		Summary		Heterogeneity	
	n^*b*^	OR	95% CI	I^2^ (%)	*p* ^*c*^
All studies	15	**0.82**	**0.72-0.93**	52.9	0.008
**Study design**					
Case-control	10	**0.81**	**0.69-0.95**	43.8	0.067
Cohort	5	0.82	0.65-1.04	53.5	0.072
**Sex**					
Female	3	**0.66**	**0.49-0.88**	8.2	0.336
Male	3	0.96	0.77-1.18	0	0.820
**Smoking status**					
Smoker	7	**0.86**	**0.74-0.99**	45.7	0.087
Non-smoker	7	1.01	0.88-1.15	37.0	0.146
**Cancer site**					
Aerodigestive tract	4	**0.67**	**0.54-0.83**	20.6	0.286
Lung	8	0.84	0.71-1.00	58.1	0.019
Stomach	3	0.95	0.67-1.35	0	0.442
Urinary organs	4	0.86	0.70-1.06	0	0.508

a Random effects model was used if studies were heterogeneous (*p* < 0.1).

b Selected study numbers

c *p* values for heterogeneity from Q-test

The summary estimates calculated for the association between the subclasses of dietary flavonoids and smoking-related cancer risk are presented in Table 4. Dietary flavonols, flavones, flavanones, flavan-3-ols, and isoflavones significantly lowered the risk of smoking-related cancers, and the significant associations were maintained among the flavones, flavanones, and isoflavones in case-control studies. Flavonols were strongly associated with lower smoking-related cancer risk. The associations with flavonols were significant in only cohort studies, and all of the flavonol subgroups were significantly associated with smoking-related cancer risk if the fixed effects model was used. The subgroup analysis conducted by smoking status showed significantly different results. The dietary intake of flavonols, flavones, and flavanones was significantly associated with a lower risk of smoking-related cancer among smokers, but no association was observed among non-smokers, except for flavanones. Quercetin and kaempferol, the components of flavonols, showed significant associations, but flavonol myricetin showed no association. However, the associations with flavonol quercetin, kaempferol, and myricetin differed by smoking status. The components of flavones (luteolin and apigenin), flavanones (naringenin), and flavan-3-ols (catechin, epicatechin) were not significantly associated with smoking-related cancer risk, but hesperetin, a component of flavanones, was positively associated with smoking-related cancer risk.

**Table 4:** Summary estimates for the effect of subclasses of dietary flavonoids on risk of smoking-related cancer^**a**^.

		Summary		Heterogeneity			Summary		Heterogeneity				Summary				Heterogeneity		
	n^*b*^	OR	95% CI	I^2^ (%)	*p* ^*c*^	n^*b*^	OR	95% CI	I^2^ (%)	*p* ^*c*^	n^*b*^		OR		95% CI		I^2^ (%)		*p* ^*c*^
	Flavonols					Flavones					Flavanones								
All studies	15	**0.77**	**0.63-0.95**	70.3	< 0.001	13	**0.77**	**0.69-0.85**	0	0.553	13	**0.77**		**0.64-0.92**		64.8		0.001	
Case-control	11	0.80	0.61-1.04	76.1	< 0.001	10	**0.73**	**0.65-0.83**	0	0.735	11	**0.74**		**0.60-0.90**		67.6		0.001	
Cohort	4	**0.77**	**0.63-0.95**	39.2	0.176	3	0.86	0.70-1.06	34.6	0.217	2	0.97		0.75-1.24		0		0.650	
Female	5	**0.66**	**0.56-0.78**	17.0	0.306	3	**0.58**	**0.44-0.77**	0	0.667	4	**0.74**		**0.62-0.88**		32.8		0.215	
Male	5	**0.88**	**0.79-0.99**	0	0.621	3	**0.79**	**0.63-0.98**	0	0.628	4	**0.81**		**0.73-0.90**		0		0.637	
Smoker	8	**0.82**	**0.71-0.95**	51.4	0.044	5	**0.82**	**0.68-0.99**	67.8	0.014	7	**0.77**		**0.68-0.89**		61.5		0.016	
Non-smoker	8	0.84	0.69-1.03	62.4	0.009	5	0.99	0.89-1.11	1.9	0.396	7	**0.77**		**0.61-0.96**		74.4		0.001	
	Flavan-3-ols					Anthocyanidins					Isoflavones								
All studies	15	**0.88**	**0.79-0.98**	16.2	0.264	12	0.89	0.79-1.01	0	0.649	11	**0.85**		**0.78-0.94**			30.7		0.146
Case-control	11	0.90	0.80-1.01	0	0.483	10	0.90	0.79-1.03	0	0.476	7	**0.75**		**0.66-0.86**			0		0.560
Cohort	4	0.71	0.45-1.12	54.0	0.089	2	0.86	0.66-1.13	0	0.847	4	0.96		0.84-1.09			0		0.424
Female	3	**0.67**	**0.51-0.90**	0	0.439	3	0.78	0.57-1.08	0	0.406	6	0.91		0.67-1.22			59.6		0.030
Male	3	1.03	0.83-1.28	0	0.551	3	0.97	0.76-1.23	0	0.996	6	0.84		0.69-1.03			51.5		0.067
Smoker	6	0.94	0.79-1.12	56.4	0.043	5	0.92	0.86-1.00	4.7	0.380	5	0.86		0.72-1.04			55.5		0.061
Non-smoker	6	0.99	0.91-1.07	15.4	0.315	5	1.33	0.95-1.88	61.3	0.035	5	0.78		0.59-1.02			55.5		0.062
	Quercetin					Kaempferol					Myricetin								
All studies	10	**0.80**	**0.67-0.96**	45.2	0.058	9	**0.85**	**0.74-0.97**	27.1	0.203	7	0.90		0.78-1.02			0		0.815
Case-control	7	**0.75**	**0.59-0.97**	51.8	0.053	6	0.80	0.61-1.07	51.0	0.070	4	0.86		0.71-1.05			0		0.604
Cohort	3	0.89	0.73-1.08	0	0.452	3	0.86	0.71-1.04	0	0.697	3	0.93		0.77-1.12			0		0.657
Smoker	3	**0.60**	**0.46-0.80**	0	0.940	2	**0.50**	**0.34-0.72**	79.3	0.028	2	**0.64**		**0.45-0.89**			0		0.571
Non-smoker	3	0.77	0.56-1.05	28.8	0.246	2	0.86	0.60-1.24	0	0.423	2	0.84		0.60-1.17			0		0.664
	Luteolin					Apigenin					Hesperetin								
All studies	3	0.99	0.76-1.29	0	0.450	1	-	-	-	-	2	**1.46**		**1.07-1.98**			0		0.390
	Naringenin					Catechin					Epicatechin								
All studies	2	0.96	0.52-1.76	85.6	0.008	3	0.74	0.53-1.03	60.0	0.082	3	0.82		0.66-1.01			4.1		0.352

a Random effects model was used if studies were heterogeneous (*p* < 0.1).

b Selected study numbers

c *p* values for heterogeneity from Q-test

Table 5 shows summary estimates for the effect of subclasses of dietary flavonoids on cancer risk by cancer site. In aerodigestive tract cancer including oral, larynx, pharynx, and esophageal cancer, most of flavonoids subclasses were significantly associated with reduced risk. In lung cancer, flavonol quercetin and kaempferol were inversely associated with cancer risk, but these associations were not observed in total flavonol intake and any other flavonoid subclasses**.**

**Table 5:** Summary estimates for the effect of subclasses of dietary flavonoids on cancer risk by cancer site^a^.

		Summary				Heterogeneity		
	**n^b^**		**OR**	**95% CI**	**I^2^ (%**)		***p*^*c*^**
Aerodigestive tract^d^							
Flavonols	4		**0.61**	**0.38-0.99**	76.9		0.005
Flavones	4		**0.81**	**0.67-0.98**	0		0.826
Flavanones	4		**0.57**	**0.43-0.76**	56.2		0.077
Flavan-3ols	4		0.84	0.68-1.04	0		0.486
Anthocyanidins	4		**0.77**	**0.62-0.97**	0		0.780
Lung							
Flavonols	3		0.93	0.45-1.89	85.7		0.001
Quercetin	5		**0.66**	**0.47-0.92**	49.6		0.094
Kaempferol	5		**0.78**	**0.64-0.95**	0		0.871
Myricetin	3		0.93	0.73-1.18	24.0		0.268
Flavanones	3		0.93	0.62-1.41	70.3		0.034
Flavan-3ols	5		0.91	0.69-1.19	52.0		0.080
Urinary organs							
Quercetin	3		0.99	0.76-1.29	0		0.465
Kaempferol	3		1.03	0.79-1.32	44.4		0.166
Myricetin	3		1.13	0.75-1.71	0		0.935
Flavan-3ols	3		0.83	0.64-1.06	0		0.460

a Random effects model was used if studies were heterogeneous (*p* < 0.1).

b Selected study numbers

c *p* values for heterogeneity from Q-test

d oral, larynx, pharynx, and esophageal cancer

Subgroup analyses including less than 2 studies were omitted.

## Discussion

Meta-analyses performed in this study including 19 case-controls (9,525 cases and 15,835 controls) and 15 cohort studies (988,082 subjects and 8,161 cases) showed various results according to flavonoid type, smoking status, and cancer site. However, overall results revealed that dietary flavonoids were inversely associated with the risk of smoking-related cancers, and the associations were more prominent among smokers.

The summary estimates suggested that total dietary flavonoids and several flavonoid subclasses were associated with reduced risks of smoking-related cancers. Total dietary flavonoids were inversely associated with smoking-related cancer risk in both the case-control and cohort studies, although a non-significant association was observed in the cohort studies. Flavones, flavanones, isoflavones, and flavonol quercetin were inversely associated with the risk of smoking-related cancers in only the case-control studies. Similar results were shown in our previous meta-analysis of colorectal and stomach cancers [9]. Significant associations were observed in the summary estimates of only the case-control studies, which are subject to more recall bias than cohort studies. However, the non-significant results might be caused by low statistical power due to the small numbers of cohort studies in the present study. In the summary estimates of cohort studies, dietary flavonols were significantly associated with a reduced risk of smoking-related cancer, and total flavonoids were also significantly associated with the reduced risk when the fixed effects model was applied. Thus, in contrast to previous study of colorectal and stomach cancers [9], dietary flavonoids may play a role in reducing smoking-related cancer risk. The preventative effects of dietary flavonoids may differ by cancer site among the smoking-related cancers. Lung cancer, which is mostly caused by tobacco smoke, was the most commonly studied cancer site, but total dietary flavonoids was only marginally associated with lung cancer in the present study. Significant association between flavonoids and lung cancer risk was observed in the previous meta-analysis [47]. In their study, summary estimate of flavonoids were combined all studies reported total flavonoid intake and their subclasses altogether. However, the results of each flavonoid subclasses were similar. Kaempferol alone showed significant association with reduced lung cancer risk in the previous meta-analysis, and quercetin, although it is not statistically significant, showed most reduced risk of lung cancer. In our study, quercetin and kaempferol were significantly associated with reduced risk of lung cancer. Intake of total dietary flavonoids and most of their subclasses was significantly associated with aerodigestive tract cancer risk. Dietary flavonoids were not significantly associated with risk of cancer at any other site. The limited number of studies that were eligible for the subgroup analyses conducted by cancer site rendered the results inconclusive. However, the lower risk of cancers of the aerodigestive tract may be closely related to the protective effects of dietary flavonoids.

The protective effects of flavonoids on cancer risk have been explained by several mechanisms. Flavonoids are polyphenolic compounds that are known to have antioxidant properties, so the free radical scavenging properties of flavonoids are closely related to the beneficial effects on cancer risk. The strong antioxidant properties of flavonoids effectively reduced various types of oxidants [48,49]. However, direct antioxidant activities of dietary flavonoids may not be the only explanation of the protective effects on cancer risk [50,51]. Dietary flavonoids are found at very low concentrations in human plasma due to their low bioavailability. The bioavailability of dietary flavonoids can vary according to their chemical structure and food sources. The bioavailability of isoflavones and quercetin was relatively high, whereas that of tea catechins and anthocyanins is very low [50,52]. The other explanation for the beneficial effects of flavonoids on cancer is that flavonoids play a role in regulating enzymatic pathways. Carcinogens that enter the body are first metabolized to more active forms by phase I enzymes such as cytochrome P450 (CYP), and the active forms can be detoxified by phase II enzymes such as UDP-glucuronyl transferase, glutathione S-transferase, and quinone reductase. Flavonoids are responsible for the inactivation of phase I enzymes as well as the activation of phase II enzymes [53].

Several studies examined for this meta-analysis performed a subgroup analysis by smoking status. Total flavonoid intake was associated with a reduced cancer risk among smokers but not among non-smokers. These different associations were observed for dietary flavonols, flavones, flavanones, and the flavonol components quercetin, kaempferol, and myricetin. Because smoking can increase oxidative stress, dietary flavonoids may function as antioxidants; therefore, the more prominent effects of flavonoids on the reduced cancer risk may be more likely to be observed among smokers. Smokers have lower levels of some antioxidants in their plasma than non-smokers, suggesting the larger involvement of these antioxidants in smokers [54]. Similar results were observed in a previous meta-analysis of dietary flavonoids and the risk of lung cancer, and the results suggested that flavonoids function as antioxidants to reduce the lung cancer risk in smokers [47]. However, the antioxidant activities of dietary flavonoids might be limited due to their low bioavailability, as discussed earlier. Tobacco has many types of carcinogens including PAH, benzo[a]pyrene (BaP), N-nitroso compound, and 4-aminobiphenyl, and these carcinogens and their metabolites can cause gene mutations such as p53 mutations and the formation of DNA or protein adducts [8]. Dietary flavonoids protect human hepatoma cells from N-nitrosodimethylamine-, N-nitrosopyrrolidine- and BaP-induced DNA damage [55]. Associations between CYP1A1 and lung squamous cell carcinoma (SCC) and associations between CYP2E1 and lung adenocarcinoma (AC) were observed, suggesting a specificity of tobacco smoke PAHs for lung SCC and tobacco-specific nitrosamines for lung AC [56]. Quercetin inhibited the CYP450 enzymes that were activated by BaP [57], and the relationship between the consumption of onions rich in flavonoids and lung cancer risk was modified by CYP1A1 [17]. Dietary flavonoids may protect against carcinogenesis by modulating enzymatic pathways, suggesting that dietary flavonoids can function as anti-tumor agents among smokers.

Flavonoids may be associated with early carcinogenesis [58], suggesting that the cohort design is more suitable for association studies, but only a limited number of cohort studies have been published. Definition of high vs. low intake of flavonoids was slightly different across studies, which may result in high heterogeneity in the meta-analysis. Bioactive food components are highly correlated, and the bioavailability of dietary flavonoids differ considerably by food source, even among the same flavonoid types. Previous work has suggested that flavonoid food sources rather than specific flavonoids should be analyzed for associations with disease risk [59]. However, consistent associations between dietary flavonoids and their subclasses and smoking-related cancer risk were observed in the studies reviewed here. Furthermore, the subgroup analysis conducted by smoking status showed a consistently inverse association between dietary flavonoids and smoking-related cancer risk. Thus, despite these limitations, the present meta-analysis showed that dietary flavonoids are inversely associated with smoking-related cancer risk.

Although the protective effects of flavonoids varied according to cancer site and flavonoid type, the overall results indicated that dietary flavonoids might have preventative effects against smoking-related cancers. The beneficial effects of flavonoids on cancer risk were more prominent among smokers than non-smokers. Total flavonols and flavonol quercetin may be the most powerful agents for smoking-induced carcinogenesis. Thus, we suggest that smokers might benefit from a flavonoid-rich diet.

## Supporting Information

Checklist S1 **PRISMA checklist.**
(DOC)
